# Immunogenic change after percutaneous microwave ablation in pulmonary malignancies: Variation in immune cell subsets and cytokines in peripheral blood

**DOI:** 10.3389/fimmu.2022.1069192

**Published:** 2022-12-09

**Authors:** Liang Zhang, Mingming Zhang, Jun Wang, Yang Li, Taijie Wang, Jianguo Xia, Bo Feng, Jialin Shen

**Affiliations:** ^1^Department of Interventional Oncology, Renji Hospital, Shanghai Jiao Tong University School of Medicine, Pudong, Shanghai, China; ^2^School of Clinical Medicine, Jining Medical University, Jining, Shandong, China; ^3^Department of Radiology, Shanghai Jiao Tong University School of Medicine, Pudong, Shanghai, China; ^4^Department of Radiology, People’s Hospital of Qintong, Taizhou, Jiangsu, China; ^5^Department of Ultrasound, Shanghai Jiao Tong University School of Medicine, Pudong, Shanghai, China; ^6^Department of Interventional Radiology, The First Hospital of China Medical University, Shenyang, Liaoning, China

**Keywords:** CD8+ T cell, IL-2, percutaneous microwave ablation, pulmonary malignancies, regulatory T cells, immune system

## Abstract

**Introduction:**

To investigate immunogenic changes after percutaneous microwave ablation (MWA) in pulmonary malignancies.

**Methods:**

Twenty-two consecutive patients with pulmonary malignancies who underwent percutaneous lung tumor MWA were prospectively enrolled in this study. Peripheral blood samples were collected on the day before (D0) and one month (M1) after MWA. Changes in immune cell subsets (CD3+, CD4+, and CD8+ T cells, and B, natural killer, regulatory T (Treg), and CD3-CD20+ cells) and cytokines (interleukin [IL]-2, 4, 6, 10, 17A, tumor necrosis factor [TNF]-α, and interferon-γ) were noted and compared. Progression-free survival (PFS) and potentially related factors were analyzed.

**Results:**

The proportion of CD8+ T cells increased from 22.95 ± 7.38% (D0) to 25.95 ± 9.16% (M1) (*p* = 0.031). The proportion of Treg cells decreased from 10.82 ± 4.52% (D0) to 8.77 ± 2.05% (M1) (*p* = 0.049). The IL-2 concentration was also decreased from 1.58 ± 0.46 pg/mL (D0) to 1.26 ± 0.60 pg/mL (M1) (*p* = 0.028). The reduction in Treg cells predicted PFS independently of clinical prognostic features in multivariate analysis (hazard ratio = 4.97, 95% confidence interval: 1.32–18.66, *p* = 0.018). A reduction in the proportion of Treg cells was observed in 15 patients (68.2%) and the average of the reduction was 2.05 ± 4.60%. Those patients with a reduction in the proportion of Treg cells that was more than average showed a significantly longer median PFS time than those with a reduction that was less than average (16 months *vs*. 8.5 months, *p* = 0.025).

**Discussion:**

Percutaneous MWA of pulmonary malignancies leads to immunogenic changes. The reduction in the proportion of Treg cells was independently associated with PFS.

## Introduction

Thermal ablation has been widely used for the local ablation of solid tumors, with the advantages of high treatment efficacies and low complication risks (1–4). An increasing number of studies have demonstrated that thermal ablation not only destroys the tumor locally, but also leads to a change in systemic anti-tumor immunity (5–7). Such a change in immunity has been shown to predict the prognosis of patients (8, 9).

Although the mechanism of how thermal ablation can activate anti-tumor immunity has not been fully elucidated, recent studies have suggested that thermal ablation could modulate systemic anti-tumor immunity by activating various steps in the cancer immunity cycle, including: release of neoantigen and danger signals (10, 11); upregulation of immune cells in peripheral blood (12, 13); increased tumor-infiltrating lymphocytes (14, 15); elevation of interferon-gamma (IFN-γ) and other pro-inflammatory cytokines (16, 17), and a reduction in immunosuppressive regulatory T (Treg) cells (18).

In recent years, thermal ablation of primary and metastatic lung malignancies has gained in popularity for patients whose tumors are not suitable for surgical resection (19–22). However, previous studies regarding the immunity change caused by thermal ablation mainly focused on hepatocellular carcinoma (HCC), and breast, renal cell, and prostate cancers. Few to no studies have investigated immunity changes after thermal ablation of pulmonary malignancies (23). Thus, in the present study, immune cell subsets and cytokines were determined in the peripheral blood of patients, and compared before and after the percutaneous microwave ablation (MWA) of pulmonary malignancies. The aim of the study was to investigate immunogenic change after percutaneous lung MWA and its relationship with the prognosis of patients.

## Methods

### Study design

This monocentric prospective study was performed at our institution from January 2020 to December 2020. This study was approved by the local Institutional Review Board and informed consent was obtained from all eligible patients. After a multidisciplinary meeting that included surgeons, interventional radiologists, pathologists and oncologists, 104 consecutive patients who were recommended to receive MWA for pulmonary malignancies were assessed. Patients aged > 18 years, with a diagnosis of primary and metastatic lung malignancies by pathology, with less than five lesions, and with tumors that had a maximum diameter ≤5cm were included. Exclusion criteria included: 1) those with contraindications of MWA; 2) received chemotherapy, immunotherapy, radiotherapy or MWA within three months before enrollment in the study or within one month after MWA; 3) experienced severe complications from MWA such as infections or massive bleeding; 4) received glucocorticoid treatment or immunosuppressive therapy; 5) declined to participate in the study.

**Figure 1:**
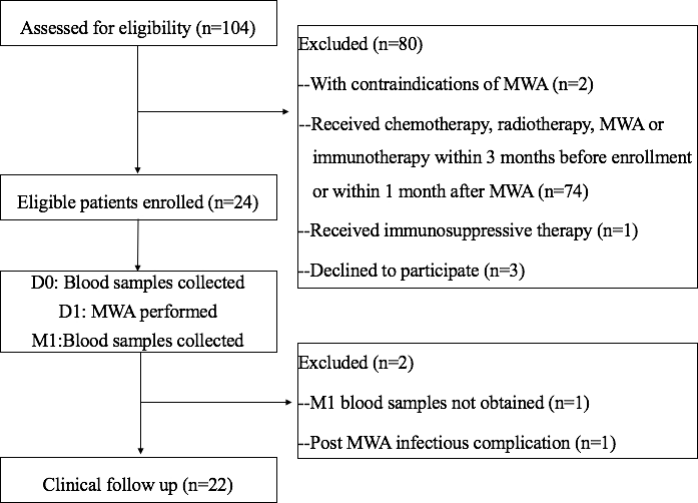
Flowchart of the study. D0, day before MWA; D1, day of MWA; M1, one month after MWA; MWA, percutaneous microwave ablation.

The day when MWA was performed was defined as D1. Patients’ peripheral blood samples were collected on the day before (D0) and one month (M1) after MWA, and were used to measure changes in immune cell subsets and cytokines. All patients received a clinical follow-up (**Figure 1**).

### Percutaneous microwave ablation

Percutaneous MWA was performed by an experienced interventional radiologist under the guidance of a dual-source computed tomography (CT) system (SOMATOM Force, Siemens Healthcare, Forchheim, Germany) with local anesthesia (lidocaine). The MWA system (MTC-3C, Vison-China Medical Devices R&D Center, Nanjing, China) consisted of a 2450 ± 50MHz generator generating a maximum of 100 W and a water-cooled antenna (outer diameter was 16G or 18G and effective length was 150 mm or 180 mm).

Tumors with a maximal diameter less than 3 cm were treated with a single applicator that was placed in the center of the tumor. To ensure the ablation range completely covered the tumors and 0.5 cm around each tumor, tumors with a diameter larger than 3 cm were treated using two to three different sites based on tumor size and shape. A second or even third ablation was performed for those with a progression in ablation lesions.

A tumor treated according to the protocol and with a patient who was completely recovered as determined at the time of the procedure was deemed a “technical success”.

All patients received a chest CT scan the next day after MWA to assess for pneumothorax or hemorrhage. Analgesics and antipyretics were prescribed if necessary.

### Measurement of immune cell subsets and cytokines

Peripheral blood samples were collected from patients on D0 and M1. T cells (CD3+), CD4+ T cells (CD3+CD4+), CD8+ T cells (CD3+CD8+), B cells (CD3-CD19+), natural killer (NK) cells (CD3-CD16+CD56+), CD20+ B cells (CD45+CD3-CD20+), and Treg cells (CD3+CD4+CD25+CD127-) were analyzed by flow cytometry (BD FACSCalibur, BD Biosciences, Sparks, MD, USA) using BD Multi-TEST IMK Kits (BD Biosciences, Shanghai, China). Levels of cytokines: interleukin-2 (IL-2), IL-4, IL-6, IL-10, IL-17A, tumor necrosis factor-alpha (TNF-α), and IFN-γ were determined by a Luminex 200 analyzer (Shanghai Tellgen Life Science Co. Ltd, Shanghai, China) using cytokine detection kits (Bio-Rad, Hercules, CA, USA).

### Treatments after MWA

For primary lung malignancies, patients with sensitive mutations were treated with epidermal growth factor receptor-tyrosine kinase inhibitors (EGFR-TKIs) or anaplastic lymphoma (ALK) kinase inhibitors. For those without mutations, platinum-based chemotherapy was administered. For patients with metastatic lung malignancies, systematic chemotherapy was administered according to the pathology of the primary tumor. For all patients, if local therapy (such as radiotherapy, a second thermal ablation, transhepatic arterial chemotherapy, and embolization) or immunotherapy was necessary, such treatments were administered one month after MWA.

### Complications and follow-up

Treatment-related complications were noted within 30 days after ablation, and were classified in accordance with the Common Terminology Criteria for Adverse Events version 4.0 (24). Patients with major complications were excluded from the study.

Patient follow-up was conducted at 1 month and every 3 months thereafter until death or the last follow-up. Follow-up evaluation included physical examination, laboratory testing, and contrast-enhanced imaging (ultrasound, CT, magnetic resonance imaging, or positron emission tomography).

Technique efficacy referred to a defined prospective time point when “complete ablation” of the macroscopic tumor was achieved as noted on follow-up imaging. Complete ablation was defined as lesion disappearance, complete cavitation formation, fibrotic progression or scar formation, solid nodule involution or no change, and/or atelectasis presenting as no contrast-enhanced signs on follow-up CT images. Incomplete ablation was indicated by incomplete cavernous formation with several remaining solid or liquid components; partial fibrosis or fibrotic lesions with solid residues; and/or solid nodules with unchanged or increased size displaying irregular peripheral or internal enhancement signs on follow-up CT images.

Treatment response was evaluated according to the Response Evaluation of Criteria in Solid Tumors version 4.0 (25). Progression-free survival (PFS), was defined as the interval between initial treatment and disease progression or death.

### Statistical analysis

Statistical software (SPSS version 19.0; SPSS, Chicago, IL, USA) was used for analysis. Continuous variables were presented as mean ± standard deviation and compared by paired sample *t* test. Categorical variables were presented as frequencies and compared using the chi-square test. Univariate and multivariate analyses were used to identify factors associated with PFS. Progression-free survival was analyzed using Kaplan–Meier curves and a log-rank test. A Cox proportional hazards model was used to examine risk factors associated with PFS. Variables with a *p* value less than 0.1 were entered into the multivariate model. The relationship among those variables was analyzed by using spearman correlation coefficients. A *p* value less than 0.05 indicated a significant difference.

## Results

### Characteristics of study population

Between January 2020 and December 2020, 104 consecutive patients were assessed. A total of 24 eligible patients were enrolled in the study. Technical success was achieved in all 24 patients. One patient was excluded due to experiencing pneumonia after MWA. Another patient was excluded due to a lack of M1 blood sample. Finally, 22 patients finished the study. **Table 1** summarizes the main characteristics of the studied population.

**Table 1:** Characteristics of study population.

Characteristics	Values
Gender (male/female)	13/9
Age (years)	67.04 ± 10.07
Histological type	
Pulmonary adenocarcinoma	13
Pulmonary squamous cell carcinoma	3
Small cell lung cancer	1
Metastatic lung cancer	5
Primary/Metastatic	17/5
Diameter of index tumor (cm)	2.38 ± 1.21
Stage	
I	3
II	2
III	4
IV	12
ECOG PS	
0	7
1	3
2	9
3	3
Other treatment after MWA	
Targeted drugs	6
Chemotherapy/TACE	6
Radiotherapy	3
No other treatment	7

ECOG PS, Eastern Cooperative Oncology Group performance status; MWA, percutaneous microwave ablation; TACE, transhepatic arterial chemotherapy and embolization.

### Variation in immune cell subsets and cytokines after MWA

**Figure 2:**
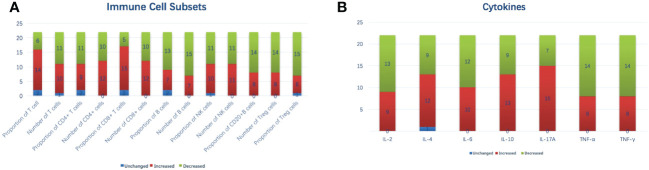
Variations in immune cell subsets **(A)** and cytokines **(B)** after MWA on a per-patient basis. IL, interleukin; MWA, percutaneous microwave ablation; NK, natural killer; TNF, tumor necrosis factor.

The variations in immune cell subsets and cytokines after MWA are shown in **Table 2** and **Figures 2A** **, B**. The proportion of CD8+ T cells increased from 22.95 ± 7.38% (D0) to 25.95 ± 9.16% (M1) (*p* = 0.031). An increase in the proportion of CD8+ T cells was observed in 15 (68.2%) patients. The proportion of Treg cells decreased from 10.82 ± 4.52% (D0) to 8.77 ± 2.05% (M1) (*p* = 0.049). A decrease in proportion of Treg cells was observed in 15 (68.2%) patients. The IL-2 concentration at M1 was significantly lower than that at D0 (1.26 ± 0.60 pg/mL *vs*. 1.58 ± 0.46 pg/mL, *p* = 0.028). A decrease in the IL-2 concentration was observed in 13 (59.1%) patients. Other immune cell subsets and cytokines showed no significant difference between D0 and M1.

**Table 2:** Variations in immune cell subsets and cytokines after MWA.

	D0	M1	Variation	P value
Immune Cells				
Proportion of T cells (%)	63.64 ± 12.53	65.68 ± 11.78	2.05 ± 6.69	0.167
Number of T cells (*10^9^/L)	796.14 ± 394.06	835.95 ± 381,73	39.82 ± 193.37	0.345
Proportion of CD4+ T cells (%)	38.86 ± 9.32	37.86 ± 10.59	-1 ± 6.33	0.467
Number of CD4+ cells (cells/uL)	485.82 ± 244.30	484.86 ± 245.62	-0.95 ± 91.48	0.961
Proportion of CD8+ T cells (%)	22.95 ± 7.38	25.95 ± 9.16	3 ± 6.07	0.031
Number of CD8+ cells (cells/µL)	286.55 ± 167.77	333.14 ± 187.46	46.59 ± 121.91	0.087
Proportion of B cells (%)	12.32 ± 8.97	10.82 ± 7.61	-1.5 ± 5.17	0.188
Number of B cells (cells/µL)	177.59 ± 177.03	155.23 ± 156.67	-22.36 ± 56.46	0.077
Proportion of NK cells (%)	22.50 ± 12.02	22.36 ± 10.37	-0.14 ± 7.86	0.936
Number of NK cells (cells/µL)	261.77 ± 161.33	299.59 ± 253.93	37.82 ± 198.29	0.381
Proportion of CD20+B cells (%)	11.71 ± 9.07	10.92 ± 8.13	-1.29 ± 5.60	0.449
Number of Treg cells (cells/µL)	46.50 ± 14.46	40.72 ± 19.22	-5.77 ± 19.82	0.186
Proportion of Treg cells (%)*	10.82 ± 4.52	8.77 ± 2.05	-2.05 ± 4.60	0.049
Cytokines				
IL-2 (pg/ml)	1.58 ± 0.46	1.26 ± 0.60	-0.32 ± 0.65	0.028
IL-4 (pg/ml)	2.02 ± 1.23	4.65 ± 7.11	2.62 ± 6.42	0.069
IL-6 (pg/ml)	16.16 ± 24.54	11.33 ± 9.38	-4.84 ± 18.45	0.233
IL-10 (pg/ml)	2.85 ± 2.20	2.80 ± 1.30	-0.06 ± 1.93	0.894
IL-17A (pg/ml)	7.34 ± 7.96	11.22 ± 11.55	3.89 ± 12.83	0.170
TNF-α (pg/ml)	1.58 ± 0.63	1.39 ± 0.84	-0.19 ± 0.89	0.343
IFN-γ (pg/ml)	2.52 ± 2.34	2.61 ± 2.69	-0.09 ± 2.79	0.881

D0: day before MWA; D1: day of MWA; IL: interleukin; IFN-γ: interferon-gamma; M1: one month after MWA; MWA: percutaneous microwave ablation; NK: natural killer cell; TNF-α: tumor necrosis factor-alpha; Treg: regulatory T cell.

*represent the proportion of Treg cells among CD4+ T cells.

### Follow-up analysis

No major post-treatment complications occurred in all patients after MWA. The average follow-up interval was 12.68 ± 7.56 (3 – 24) months. Technical efficacy was achieved in 16 patients (72.7%) during follow-up. One patient was lost to follow-up 15 months after MWA without disease progression. During the follow-up, a total of 16 patients (72.7%) showed disease progression.

In a Cox regression analysis of variables potentially associated with PFS (**Table 3**), gender (hazard ratio [HR] = 0.22, 95% confidence interval [CI]: 0.06–0.82), stage (HR = 4.26, 95% CI: 0.88–20.71), PS (HR = 1.98, 95% CI: 1.15–3.42), technical efficacy (HR = 0.31, 95% CI: 0.10–0.97), and a reduction in the proportion of Treg cells (HR = 3.30, 95% CI: 1.06–10.25) were significantly associated with PFS in univariate analysis. No linear correlation was found among the variables that entered into the multivariate model according to spearman correlation analysis (all p > 0.05). In multivariate analysis, a reduction in the proportion of Treg cells (HR = 4.97, 95% CI: 1.32–18.67, *p* = 0.018) was independently associated with PFS.

**Table 3:** Univariate and multivariate Cox regression analyses of variables potentially associated with PFS.

Variables	Patients	Univariate analysis			Multivariate analysis		
		HR	95% CI	*P*-value	HR	95% CI	*P*-value
Age		0.88	0.33-2.93	0.807			
≤ 60 years	9						
>60 years	13						
Gender		0.22	0.06-0.82	0.024	3.19	0.71-14.29	0.129
male	13						
female	9						
Diameter of index tumor		2.71	0.80-9.21	0.109			
≤ 3 cm	18						
>3cm	4						
Histological type		1.28	0.87-1.88	0.203			
pulmonary adenocarcinoma	13						
pulmonary squamous cell carcinoma	3						
small cell lung cancer	1						
metastatic lung cancer	5						
Primary		0.49	0.157-1.53	0.218			
Yes	17						
No	5						
Stage		4.26	0.88-20.71	0.073	5.15	0.52-51.23	0.162
I	3						
II	2						
III	4						
IV	12						
PS		1.98	1.15-3.42	0.014	1.44	0.76-2.75	0.264
0	7						
1	3						
2	9						
3	3						
Other treatment		2.77	0.78-9.77	0.114			
Yes	15						
No	7						
Technical efficacy		0.31	0.10-0.97	0.044	0.73	0.21-2.56	0.62
Yes	16						
No	6						
Increase in CD8+ T cell proportion		1.62	0.52-5.03	0.404			
≥ average	9						
<average	13						
Reduction in Treg cells proportion		3.302	1.06-10.25	0.039	4.97	1.32-18.67	0.018
≥ average	8						
<average	14						
Reduction in IL-2 level		0.43	0.15-1.25	0.122			
≥ average	11						
<average	11						

CI, confidence interval; HR, hazard ratio; IL, interleukin; PS, performance status; Treg, regulatory T cell.

**Figure 3:**
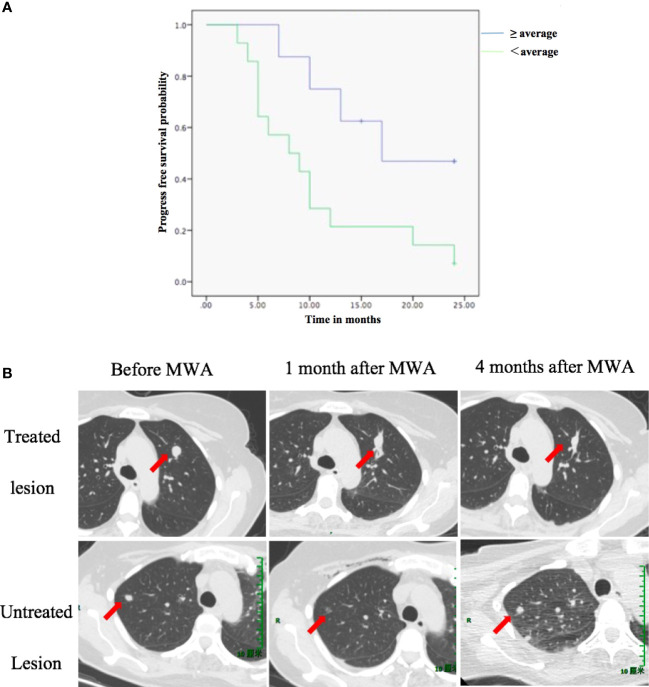
The reduction in the proportion of Treg cells is associated with PFS. **(A)** PFS according to a reduction in the proportion of Treg cells between D0 and M1. **(B)** A case showing that a reduction in proportion of Treg cells predict PFS. A 49-year-old female was diagnosed with pulmonary oligometastasis of urothelial carcinoma and underwent MWA (upper row: arrow indicated the change of treated lesion at different time point). One month after ablation of the index tumor, an abscopal effect was observed: untreated tumor regression (lower row: arrow indicated the change of untreated lesion at different time point). At M1, the proportion of CD8+ T cells increased from 39% (D0) to 53% (M1). However, the proportion of Treg cells also increased from 8.1% (D0) to 9.7% (M1). Finally, tumor progression was observed 4 months after MWA. The patient’s overall survival time was 10 months. D0, day before MWA; M1, one month after MWA; MWA, percutaneous microwave ablation; PFS, progression-free survival; Treg, regulatory T cell.

Patients with an absolute percentage decrease in the proportion of Treg cells over the average showed longer PFS (median PFS: 16.0 months) than patients with an absolute percentage decrease below the average (median PFS: 8.5 months) (**Figure 3A**, log-rank test: *p* = 0.025. **Figure 3B**, showed a case of a reduction in proportion of Treg cells predict PFS).

## Discussion

The present prospective study investigated immunogenic changes after percutaneous lung MWA and analyzed potentially related factors associated with patients’ prognosis. Our data indicated that percutaneous MWA of pulmonary malignancies leads to an immunogenic change, including an increase in the proportion of CD8+ T cells and a decrease in the proportion of Treg cells and IL-2 concentration. The reduction in the proportion of Treg cells was independently associated with PFS.

Several studies have investigated the relationship between thermal ablation of lung tumors and systemic anti-tumor immunity. Fietta et al. (13) reported that 30 days after radiofrequency ablation (RFA) of lung tumor, a significant reduction in Treg cells count with an increase in CD4+ T cell proliferation and number of IFN-γ–secreting cells was observed. The reduction in Treg cells lasted up to 90 days after treatment. Schneider et al. (26) showed that treatment of patients with RFA led to an activated and highly T-cell–stimulatory phenotype in dendritic cells, and that the RFA-induced necrotic tumor debris can serve as an *in-situ* antigen source to induce an autologous anti-tumor immune response. Wang et al. (27) reported that after RFA, the level of Th1 and Th1/Th2 cells increased, whereas levels of Th2, Th17, and Treg cells declined, indicating an improvement in anti-tumor immunity. Our data reinforce current evidence that a thermal ablation of lung tumor can trigger systemic immunological effects.

Preclinical studies have shown that lymphocytes that infiltrate the tumor as a result of thermal ablation are predominantly CD4+ and CD8+ T cells (15, 28). However, an increase in T-cell subsets was not very marked in peripheral blood samples. Zhou et al. (29) reported that levels of circulating T-cell subsets, except for Th17 cells, were relatively stable after MWA in patients with HCC. In the present study, only the proportion of CD8+ T cells increased after MWA. The amount of CD8+ T cells was not increased significantly, indicating that the change in T-cell subsets induced by MWA may be mild or transient. Other studies (18, 30) also reported that NK cell and macrophage infiltration appears to increase with thermal ablation. However, in our study, a significant change in NK and B cells was not detected. A decline in Treg cells post-ablation has been reported in several studies (13, 27). Treg cells could facilitate tumor progression by suppressing the antitumor immune response, and was reported negatively correlated with survival (31, 32). A higher number of CD4+ and CD8+ T cells and a lower number of Treg cells post-thermal ablation have been shown to have a positive effect on tumor progression and survival (33, 34). In the present study, the reduction in Treg cells was the only feature that predicted PFS independently in multivariate analysis, indicating that the MWA-induced reduction of Treg cells plays an essential role in the enhancement of systemic anti-tumor immunity.

Changes in cytokines after thermal ablation have also been reported in several studies. Elevated IFN-γ levels post-ablation has been shown to be associated with tumor-infiltrating lymphocytes (35, 36). The pro-inflammatory cytokines, IL-1β, IL-6, and IL-8, have also been noted to be elevated post-thermal ablation (17, 37). These cytokines have been shown to assist with T-cell proliferation and trafficking (38). However, in the present study, only a decrease in IL-2 concentration was found. Interleukin-2 plays an important role in the immune system by binding to the IL-2 receptor (IL-2R) on the surface of lymphocytes. It has the ability to activate CD8+ T and NK cells, and promote their proliferation. However, IL-2 can also activate Treg cells, which inhibits the anti-tumor immune response of T cells (39). The IL-2R is another key point in the process; high affinity IL-2R (complex of α/β/γ) is expressed on Treg cells, while low affinity IL-2R (complex of β/γ) is expressed on CD8+T and NK cells. Therefore, the trimer high affinity complex on Treg cells consumes a large amount of IL-2, which requires a higher dose of IL-2 to achieve cytotoxic T lymphocyte (CTL)–mediated anti-tumor immunity (40, 41). Today, research on IL-2 therapy focuses on how to enhance the activation of IL-2 on CTLs and reduce or block the activation of Treg cells (42, 43). In the present study, an increase in proportion of CD8+ T cells and a decrease in proportion of Treg cells and IL-2 concentration were observed at the same. Thus, it is postulated that MWA may induce a change in IL-2 bias; that is, IL-2 may selectively bind IL-2R on effector T cells, and reduce the binding of IL-2R on Treg cells. This hypothesis is worthy of further investigation in order to explore the potential of the combination of MWA and IL-2 therapy. Previous studies have shown that the combination of radiotherapy or PD-L1 blockade and IL-2 therapy potently stimulates systemic anti-tumor immunity (44, 45).

Several limitations exist in the present study. First, the study population was strictly selected and quite small. It may give rise to bias in statistical analysis, and the reliability of study result needs to be confirmed by further research. Second, only changes in immune cells and cytokines on D0 and M1 were analyzed. Further time points, such as an early (Day 1, Day 7) change and long term (M3, M6) change of immunity should be further investigated. Third, the mechanism of how MWA regulated immune cells and cytokines was not investigated in the present study; however, it is worthwhile to further explore the regimen in order to amplify the anti-tumor immunity induced by thermal ablation. Fourth, as the only biological material studied was blood, it is not possible to know if the observed variations were due to tumor infiltration or solely due to blood dynamics. Finally, only PFS and potentially related factors were analyzed in this study. In a further study, long-term follow-up should be conducted and overall survival should also be analyzed.

In conclusion, our study demonstrated that percutaneous MWA of pulmonary malignancies leads to immunogenic changes. The reduction found in the proportion of Treg cells was independently associated with PFS.

## Data availability statement

The raw data supporting the conclusions of this article will be made available by the authors, without undue reservation.

## Ethics statement

The studies involving human participants were reviewed and approved by Ethic Committee of Renji Hospital, School of Medicine, Shanghai Jiao Tong University. The patients/participants provided their written informed consent to participate in this study.

## Author contributions

LZ, BF and JS contributed to conception and design of the study. MZ performed statistical analysis and wrote sections of the manuscript. JW wrote the first draft of the manuscript. LZ, JS and JX performed MWA. TW and YL performed the follow-up. All authors contributed to manuscript revision, read, and approved the submitted version.
